# The Role of Irisin in Cancer Disease

**DOI:** 10.3390/cells10061479

**Published:** 2021-06-12

**Authors:** Agnieszka Pinkowska, Marzenna Podhorska-Okołów, Piotr Dzięgiel, Katarzyna Nowińska

**Affiliations:** 1 Department of Anatomy, Department of Human Morphology and Embryology, Wroclaw Medical University, 50-368 Wroclaw, Poland; agnieszka.pinkowska@umed.wroc.pl; 2 Department of Ultrastructure Research, Wroclaw Medical University, 50-368 Wroclaw, Poland; marzenna.podhorska-okolow@umed.wroc.pl; 3 Department of Histology and Embryology, Department of Human Morphology and Embryology, Wroclaw Medical University, 50-368 Wroclaw, Poland; piotr.dziegiel@umed.wroc.pl; 4 Department of Physiotherapy, University School of Physical Education, 51-612 Wroclaw, Poland

**Keywords:** Irisin, FNDC5, cancer, proliferation, migration, epithelial–mesenchymal transition

## Abstract

Irisin (Ir) is an adipomyokine that is involved in the regulation of metabolic processes. It also influences processes related to inflammation, including cancer. Initially, Ir was considered a hormone secreted by skeletal muscles in response to physical exercise. Further studies showed that Ir is also present in other healthy tissues, organs, and plasma. It influences the change in phenotype of white adipose tissue (WAT) into brown adipose tissue (BAT). It increases mitochondrial biogenesis and affects the expression of thermogenin (UCP1). This adipomyokine has also been found in many tumor tissues and in the serum of cancer patients. Studies are underway to determine the association between Ir and carcinogenesis. It has been confirmed that Ir inhibits in vitro proliferation, migration, and invasion. It is involved in the inhibition of epithelial–mesenchymal transition (EMT). Additionally, Ir affects the expression of the transcription factor Snail, which is involved in EMT, and inhibits transcription of the gene encoding E-cadherin, which is characteristic of epithelial-derived cells. Many studies have been performed to determine the role of Ir in physiological and pathological processes. Further detailed studies should determine more precisely the effect of Ir on the body in health and disease.

## 1. Introduction

Irisin (Ir) was first described in 2012 as a hormone released into the blood by skeletal muscles in response to physical exercise. In their study, Boström et al. [1] found that an increase in the expression of fibronectin type III domain-containing protein 5 (FNDC5, a membrane protein) occurs under the influence of physical exercise in the muscle tissue. Further transformation of FNDC5 results in the formation of a new protein, known as Ir. This process is controlled by the transcriptional coactivator peroxisome proliferator-activated receptor gamma coactivator 1 alpha (PGC1α). Boström et al. [1] assumed with high probability that other tissues could also be involved in the secretion of Ir. Multidirectional studies on Ir confirmed the primary assumptions of its discoverers. In addition to the primary localization, the expression of Ir has been found in other tissues and organs, i.e., in the adipose tissue [2,3], cardiomyocytes [4], kidney [4], liver [4], skin [4], and cerebellum [5].

**Figure 1:**
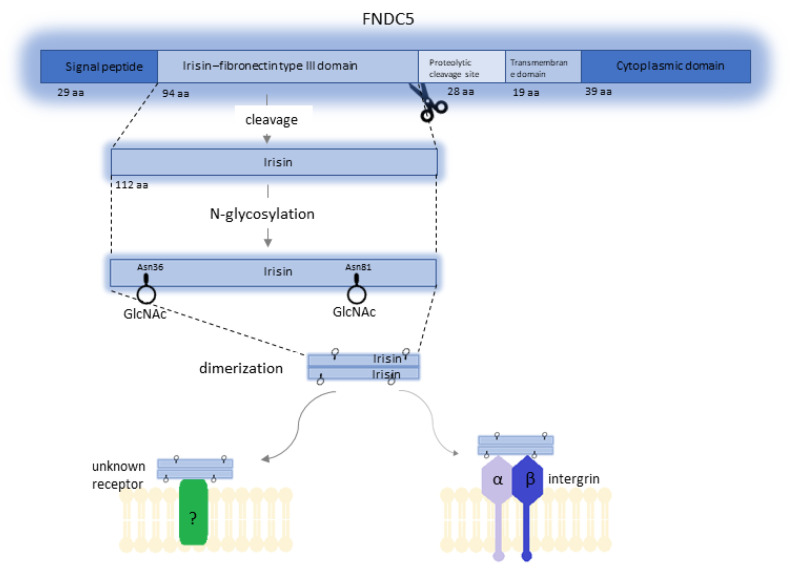
Structure of FNDC5 and Irisin. FNDC5 is composed of a 29-amino-acid signal peptide, a 94-amino-acid fibronectin type III domain, a 28-amino-acid domain (potential site of proteolytic cleavage), a 19-amino-acid transmembrane domain, and a 39-amino-acid cytoplasmic domain. The diagram also shows the glycosylation site of Irisin by the formation of a bond between *N*-acetylglucosamine and the nitrogen originating from the amide group of asparagine (Asn36 and Asn81) and subsequent dimerization of Irisin molecules. Irisin is a ligand for the integrin receptor. Perhaps, it also works by attaching to another unknown membrane receptor.

FNDC5 is the precursor of Ir. In humans, this prohormone is encoded by the *FNDC5* gene, which is located on chromosome 1 at position 35.1 (1p35.1). The *FNDC5* gene consists of six exons and five introns and spans 8.47 kbp [6]. Expression of this gene occurs under the influence of peroxisome proliferator-activated receptor gamma coactivator 1α (PGC1α), and the FNDC5 protein (also known as Frcp2 and PEP) is the product of its expression (Figure 1). The mass of the FNDC5 protein ranges from 20 to 32 kDa, and this difference is related to post-translational modification [1]. FNDC5 is composed of a 29-amino-acid signal peptide, a 94-amino-acid fibronectin type III domain, a 28-amino-acid portion of unknown function (most likely Ir proteolytic cleavage site), a 19-amino-acid transmembrane domain, and a 39-amino-acid cytoplasmic domain [6,7].

Post-transcriptional processing of FNDC5 results in the formation of four Ir isoforms via alternative splicing. Isoform 1 (Q8NAU1-1) is used as a canonical sequence. The other three isoforms, isoform 2 (Q8NAU1-2), isoform 3 (Q8NAU1-3), and isoform 4 (Q8NAU1-4), have missing sequences of amino acids at positions 182–212, 1–75, and 1–75, respectively [8]. In addition, biochemical studies have shown that Ir can exist as a homodimer, and the FNIII-like domain forms a continuous intersubunit β-sheet dimer [9]. Ir is formed as a result of further modifications due to cleavage and glycosylation of the extracellular domain of FNDC5 and contains 112 amino acids [1]. The molecular mass of Ir is estimated at 12 kDa, although studies suggest that glycosylated Ir ranging from 20 to 32 kDa is also secreted [3]. Initial studies showed remarkable conservation of Ir in mammals, which, according to Boström et al. [1], implied its conserved function mediated by a cell surface receptor. Further studies showed that human *FNDC5* is a gene with an atypical start codon to ATA (encoding isoleucine) instead of ATG (encoding methionine), which is present in the gene in animals. According to these researchers, using this noncanonical start site is associated with the generation of full-length protein, whereas the start of translation at the ATG start codon results in the formation of a truncated isoform of Ir [10]. The occurrence of Ir, which is secreted by skeletal muscles into human and mice plasma as a result of physical effort, and its potential influence on metabolism have generated much controversy. The criticism was mostly related to the research methods. Erikson [7] indicated that several studies used antibodies that were irrelevant to Ir. Boström et al. [1] used a polyclonal antibody against a peptide corresponding to C-terminal amino acids of the human FNDC5 (transmembrane segment) with no sequence from the Ir peptide. Similar objections were reported by Albrecht et al. [11] who paid attention to the variety of available assays for detecting and quantifying serum Ir. Due to their use, many studies were published in which serum Ir levels ranged from 0.01 to over 2000 ng/mL. Albrecht et al. [11] demonstrated that the antibodies they analyzed in their study showed clear cross-reactions with proteins other than Ir. Considering these concerns, some doubts occurred whether Ir was released into the plasma following physical effort and whether it could be assigned physiological functions. Jędrychowski et al. [12] performed the identification and quantitative assessment of human Ir. Studies using mass spectrometry (MS) showed that human Ir was present and was secreted into the circulation in association with physical effort. The quantitative levels of human Ir circulating in the blood were also determined. Consideration was given to the differences in concentration levels related to sedentary individuals (~3.6 ng/mL) and those who underwent training (~4.3 ng/mL). The analysis using mass spectrometry was also performed by Albrecht et al. [11] who detected a peptide that corresponded to FNDC5 or Ir. Of note, its apparently low level at the detection limit of the tested antibodies makes a physiological role of Ir very unlikely

In addition to the reservations of Albrecht et al. [11], our doubts were also raised by the presentation of the study results. Serum Ir levels of healthy controls and cancer patients were presented in different units, i.e., μg/mL [13], pg/mL [14], and ng/mL [15]. Such inconsistency makes it difficult to compare the results of different research teams. The significant distribution of Ir levels may be influenced by the selection of the study group in terms of the physical activity of the subjects, type, body shape, comorbidities, and the way of sample collection and storage, which could be an additional factor affecting the stability of serum Ir.

Many papers showed that Ir level increases with physical effort [1,16,17,18]. However, other studies did not show a strong relationship between physical effort and the level of circulating Ir [19,20,21]. There are also papers showing a baseline difference in Ir levels between physically active and inactive subjects [12], as well as between trained and untrained individuals [22]. The form and the duration of physical exercise are also of significance. Significant increases in serum Ir were found in response to vigorous physical effort after 1 week of training. However, no effect of vigorous physical exercise was reported after 8 weeks of training [16]. The analysis of 74 studies from the MEDLINE database conducted by Fatouros [23] showed that strength and endurance exercise represented a potent stimulus for release of Ir if this exercise was characterized by adequate intensity and duration. Animal studies suggested that Ir levels could also increase in response to systematic training of low intensity.

However, most human studies have produced contradictory results. According to the above research, the results might also be affected by the methodology of Ir measurement, age of subjects, their conditioning status, and exercise intensity. The half-life and physicochemical properties of Ir are determined by post-translational modifications, including glycosylation. Glycosylation is one of the most common post-translational modifications, and most membrane and secretory proteins undergo such modification. It involves the enzymatic attachment of sugar residues via a glycosidic bond [24]. Tissue-specific and cell-specific enzymes of the endoplasmic reticulum and the Golgi apparatus participate in the glycosylation process. Proteins can be modified in the process of *N*-glycosylation and/or *O*-glycosylation [25]. The *N*-glycans and *O*-glycans formed by glycosylation affect the physicochemical properties of proteins, which determines their role in metabolic processes [26]. *N*-Linked glycoproteins are created by the formation of a glycosidic bond between *N*-acetylglucosamine (GlcNAc) and the nitrogen originating from the amide group of asparagine (Asn) in the sequence Asn–X–Ser/Thr (X being any amino acid except proline) [24,25]. In oncogenesis, abnormal glycosylation is one of the key factors in tumor development. Glycopeptides are involved in cancer cell signaling, migration, invasion, and metastasis. In addition, they are involved in the relationship between the cell and the extracellular matrix. They participate in angiogenesis and influence immune cells [27]. The process of glycosylation of Ir is poorly understood. Nie et al. [28] showed that FNDC5 is an *N*-glycan with two potential glycosylation sites (Asn36 and Asn81). In the same study, they observed that inhibition of glycosylation decreased Ir secretion, reducing the stability of FNDC5 and its half-life [28]. Glycosylation also increases the molecular mass of Ir [29]. Many research teams attempted to determine the role of Ir in metabolic processes, its impact on tissues, and its potential influence on carcinogenesis. These processes may depend on the course of Ir glycosylation. Different and sometimes contradictory study results related to the effect of Ir on cells in the vitro model may also depend on whether glycosylated or non-glycosylated Ir was used in studies. The comparison of study results of selected research teams (Table 1) shows that researchers did not always accurately determine the form of Ir. Ganon et al. [30] and Shi et al. [31] indicated that such a distinction is essential for interpreting study results.

## 2. Irisin as a Ligand for Integrins

Due to the short half-life of Ir in the serum (about 20 min), Boström et al. [1] suggested that Ir could interact through a cell surface receptor. The ability of Ir to form homodimers [9] supports this hypothesis. Many studies show Ir as a protein that transfers information between the muscle tissue and other tissues. Its role ideally corresponds to its name, which is derived from the Greek goddess Iris, who was the messenger of the gods. Ir was shown to increase the mass of bone [32]. It prevents bone mass loss and influences bone healing in mice [33]. It also positively affects the mechanisms responsible for bone metabolism in mice [34]. Research showing the effects of Ir on bone tissue resulted in a study on its receptor.

Integrins are transmembrane proteins composed of α/β heterodimers, which are bidirectionally activated by the cell membrane. Physiological processes involved in integrin activation and ligand attachment determine cellular homeostasis. Abnormal activation under pathological conditions allows cell migration to tissues and the extracellular matrix, which initiates inflammatory processes and carcinogenesis [35].

Kim et al. [36] described the Ir receptor as a subset of integrin complexes. Ir was bound to several integrin complexes and showed the highest affinity for αV/β5 integrin, which was also confirmed by hydrogen–deuterium exchange/mass spectrometry (HDX/MS). Furthermore, Ir activated integrin receptor-specific signaling, including focal adhesion kinase (FAK), within 1 min of Ir being added to osteocytes. Moreover, integrin inhibitors or antagonistic antibodies directed against integrin αV/β5 inhibited Ir signaling and its further gene expression. Kim et al. [36] demonstrated that the Ir receptor formed a complex of integrins, especially those containing αV integrin, at least in osteocytes and the adipose tissue. Estel et al. [37] also showed that α_V_β_5_ integrins acted as a receptor for Ir on osteocytes. The expression of α_V_β_5_ subunits increased in osteoclast cultures after administration of Ir, and blocking the integrin complex with a neutralizing antibody completely suppressed the activating effect of Ir on osteoclastogenesis. Oguri et al. [38] showed that CD81 formed complexes with α_V_β_1_ and α_V_β_5_ integrins, mediating the activation of integrin–FAK signaling in response to Ir. CD81 molecules are markers of adipocyte progenitor cells (APCs) and are involved in cold-induced brown adipose tissue lipogenesis, and their loss causes glucose intolerance and insulin resistance. Bi et al. [39] found that Ir restored the intestinal barrier function, which is lost due to ischemia, by binding to the α_V_β_5_ integrin receptor and activating the AMPK–UCP2 pathway. In the same study, immunofluorescence staining revealed the colocalization of Ir and α_V_β_5_ integrin after administration of Ir to cells under hypoxia/reoxygenation conditions. According to Park et al. [35], the above studies demonstrate that Ir is an α_V_β_5_ ligand and, thus, exerts its effects on tissues. However, further studies are warranted to identify other membrane receptors for Ir.

## 3. Irisin as a Coordinator of Metabolic Processes

Exercise-induced Ir results in changes in the white adipose tissue (WAT) and induces browning [1]. WAT, as an energy reservoir, is mainly a store of triglycerides, whereas brown adipose tissue (BAT) is responsible for energy expenditure [26]. Uncoupling is a process in which energy is released during the oxidation of respiratory substrates in mitochondria. The uncoupling protein (UCP), also known as thermogenin, is a specific marker of BAT. UCPs are present in the inner mitochondrial membrane of all eukaryotic cells and are protein complexes that function as proton pumps. By dissipating energy and releasing it in the form of heat, UCP1 participates in the control of cell energy metabolism [40]. The function of BAT is based on the conversion of energy provided with food into heat energy.

Heat generation, known as adaptive non-shivering thermogenesis, is controlled by the adrenergic system and is related to the adaptation to life in cold climate conditions in the case of hibernating and newborn mammals, including human neonates. Under certain conditions (e.g., reduced temperature), it may also serve as a tool for regulating metabolism. In active BAT, large amounts of glucose and lipids are combusted, and the energy is dissipated in the form of heat. This is of great physiological importance to the body due to its potential role as a natural mechanism for weight control [41]. BAT has a beneficial effect on metabolism, whereas the traditionally perceived role of WAT is related to energy storage and fatty-acid release. However, its metabolic function in the body is more complex. WAT is essential for normal glucose homeostasis. It is involved in producing proinflammatory cytokines, some of which are involved in lipid metabolism, while others are involved in vascular homeostasis. Its hormonal activity (secretion of leptin, adiponectin, angiotensin, IL-6, resistin, and TNF-α) induces insulin resistance, which is responsible for the development of type II diabetes, thus linking diabetes to obesity. It promotes the development of polymetabolic syndrome, hypertension, and hypercholesterolemia, which results in cardiovascular complications, and it promotes cancer formation [42,43].

Transcription factors of the peroxisome proliferator-activated receptor (PPAR) family are involved in adipose tissue differentiation. The participation of PGC1α (a coactivator of PPARγ-1 α), which is a transcription factor that controls UCP1 expression, is required for BAT formation. PGC1α is induced in the muscle tissue by physical exercise. Boström et al. [1] conducted a study that showed WAT browning (increase in UCP1 mRNA expression of adipose cells) in mice subjected to physical activity. The results were confirmed in vitro using media conditioned by myocytes expressing PGC1α. Further studies showed an increase in FNDC5 mRNA expression, which was induced by exercise and a significant increase in UCP1 mRNA induction in BAT cell cultures under the influence of FNDC5 compared to the osteogenic protein (BMP-7), which was previously considered an inducer of browning. Physical exercise also affects the hippocampus by regulating the expression of the brain-derived neurotrophic factor (BDNF). This factor is a regulator of neuronal survival and neurogenesis in adults [44].

Wrann et al. [45] showed that PGC1α overexpression increased *FNCD5* gene expression in neurons. The increase in *FNDC5* expression depends on the formation of the PGC1α transcriptional complex with estrogen-related receptor alpha (ERRα). The researchers found that the expression of *BDNF*, *FNDC5*, and *ERRα* increased in the hippocampus due to exercise and showed that FNDC5 was a regulator of *BDNF* gene expression in neurons. Furthermore, they showed that BDNF in a feedback loop negatively regulated *FNCD5* expression. Immunohistochemical (IHC) studies also showed an increase in UCP1-positive adipocytes due to FNDC5 [1].

WAT browning induces the formation of the beige adipose tissue, which shows UCP1 expression and phenotypically and functionally resembles BAT. It is involved in controlling body temperature. It influences glucose and lipid metabolism, as well as energy homeostasis. Additionally, it has endocrine functions [46]. Ir, which is secreted in response to physical effort and stimulates an increase in UCP1 expression and its metabolic sequelae, may play an essential role in maintaining metabolic homeostasis of the body. It improves glucose homeostasis, lipid profile, and metabolic parameters. It is a promising predictive marker of insulin resistance [47]. The effect of Ir on diseases associated with polymetabolic syndrome has been widely studied, and its detailed presentation is beyond the scope of this paper.

## 4. Irisin in Cancer Proliferation Process

The aim of cancer cells is to form a tumor and create expansive forms capable of metastasizing to other areas of the body. This is possible due to the potential of tumor cells for unlimited growth, angiogenesis, or the inhibition of apoptosis. Many changes occur in tumor cells during neoplastic transformation. These changes determine growth and division and occur in the tumor microenvironment [48]. Tumor cells are characterized by increased metabolism. Rapidly proliferating normal cells and cancer cells prefer anaerobic harvesting of energy, converting glucose to lactate, even in the presence of oxygen. This phenomenon is known as the Warburg effect [49]. An alternative way of obtaining energy is provided by activated carcinoma-associated fibroblasts (CAFs) in the tumor stroma. Under oxidative stress, they provide tumor cells with the necessary substrates for anabolic processes using the Warburg effect. Due to the supplied substrates, cancer cells produce energy, mainly through aerobic respiration. This phenomenon is known as the reverse Warburg effect [50]. Ir influences cancer cell proliferation. Nowinska et al. [51] showed that Ir expression was higher in stromal cells of non-small-cell lung cancer (NSCLC) and increased in tumors with higher malignancy and higher staging, which could affect the proliferation of NSCLC cancer cells.

The energy that is produced in the process of cellular respiration is necessary for cell growth, migration, and differentiation, as well as the maintenance of constant body temperature. Glucose is the essential substrate for these processes. Due to proto-oncogene mutations and altered signaling pathways, tumor cells inhibit differentiation and use increased glucose requirements mainly for survival, growth, and proliferation [50]. The anaerobic respiration and high energy demand of tumor cells result in enhanced glycolysis and increased glucose uptake by the cells, which is mediated by glucose transporters known as GLUTs. Overexpression of membrane glucose transporters, including GLUT1, has been observed in many malignancies, including breast, colorectal, salivary, and gastric cancers [52]. Serine threonine kinase Akt, which is the main effector of phosphatidylinositol 3-kinase PI3K, plays an essential role in modifying cancer cell metabolism. Akt affects GLUT1 expression through activation of mammalian target of rapamycin kinase (mTOR kinase) [53]. Previous studies on mice showed that Ir increased glucose tolerance and uptake, as evidenced by GLUT4 translocation in skeletal muscle of diabetic mice. Ir enhanced glucose utilization by increasing 5’AMP-activated protein kinase (AMPK) phosphorylation in myocytes and hepatocytes of diabetic mice in in vitro and in vivo studies [54]. Uncontrolled tumor growth results in impaired blood supply and causes hypoxia. Hypoxia-induced factors are produced under hypoxic conditions, including the protein HIF-1α, which mediates many adaptive responses aimed at cell survival. HIF-1α increases vascular endothelial growth factor (VEGF) expression by affecting the intensification of neoangiogenesis and increased vascular permeability in the tumor. Under anaerobic conditions, HIF-1α promotes the activation of oxygen-independent metabolic pathways, including glycolysis by stimulating the expression of glucose transporters, which are crucial for increased glucose uptake [55].

**Figure 2:**
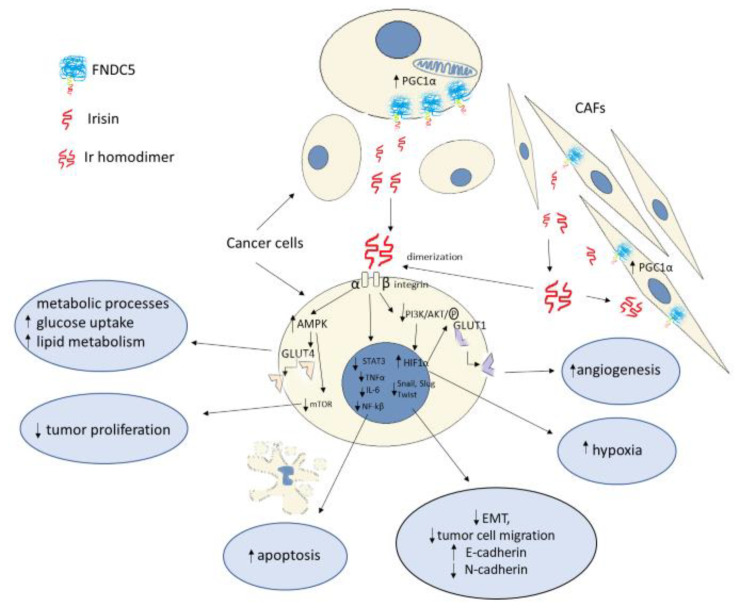
Potential roles of Irisin (Ir) in different signaling pathways and molecular processes involved in cancer progression, proliferation, angiogenesis, apoptosis, hypoxia, metabolic changes, and epithelial–mesenchymal transition (EMT) and migration of cancer cells. Ir has been observed in cancer cells and is also expressed in cancer-associated fibroblasts. This protein is cleaved from FNDC5 prohormone and has the potential to affect the neighboring cells (paracrine) or the cells from which it has been released (autocrine). Ir dimerizes and creates a homodimer with a beta sheet positioned between monomers and binds as a ligand to the integrin receptor. Ir binding to the receptor can affect many signaling pathways. Ir inhibits proliferation via the AMPK–mTOR pathway and increases glucose uptake via GLUT4 incorporation into the cell membrane. Ir affects the STAT3/Snail pathway, inhibits IL-6, and reverses EMT. Ir decreases the expression of N-cadherin and increases the expression of E-cadherin. Snail is downregulated by Ir via decreased phosphorylation of PI3K/Akt.

Activation of the PI3K/Akt pathway in an mTOR-dependent or -independent manner influences an increase in HIF-1α expression [53]. Gaggini et al. [56] showed that FNDC5 mRNA overexpression in hepatocellular carcinoma (HCC) cells was associated with increased gene expression of mediators of lipogenesis, transcription factors involved in tumorigenesis, and proinflammatory cytokines, including TNF-α and IL-6. It was also found that, in patients with HCC and tumor-enhanced lipogenesis, increased paracrine production of FNDC5/Ir could compensatively inhibit lipid synthesis. Altay et al. [57] reported a significantly increased FNDC5 expression in WAT and BAT in mice with induced gastric cancer. Cancer development corresponded to an increase in FNDC5 expression in the adipose tissue. In addition, a significant increase in serum Ir levels was demonstrated in unhealthy mice. The increase in serum Ir levels was accompanied by increased levels of TNF-α and IL-6. It was also noted that increased Ir levels in the adipose tissue could result in excess weight loss and cachexia in mice. In their in vitro study, Gannon et al. [30] demonstrated the inhibitory effect of Ir on the population size and migratory capacity of malignant breast cancer cell lines. Furthermore, they demonstrated that Ir induced apoptosis of malignant cells by inhibiting nuclear factor kappa-light-chain-enhancer of activated B cells (NF-κB) activity. This may indicate a potential anti-inflammatory effect of Ir against proinflammatory cytokines (i.e., TNF-α). The inhibitory effect of Ir on cancer cell proliferation has also been demonstrated in other studies. Tekin et al. [58] found the antiproliferative effect of Ir on prostate cancer cells in their in vitro study. Additionally, under cell culture conditions, Shao et al. [59] and Fan et al. [60] demonstrated the antiproliferative effect of Ir on lung cancer cells. Kong et al. [61] reported the inhibitory effect of Ir on the proliferation of osteosarcoma cells, whereas Liu et al. [62] reported this effect on pancreatic cancer cells. In addition to HIF-1α, other proinflammatory factors, transcription factors (NF-κB, STAT3, TNF-α, IL-6), and chemokines promote tumor proliferation by enhancing cancer cell survival, stromal remodeling, angiogenesis, and the metastatic process. The process of cancer transformation also depends on the inhibition of apoptosis, which is manifested by decreased expression of the tumor suppressor gene p53 [53]. Apoptosis is also influenced by Akt, which directly participates in the phosphorylation of proapoptotic proteins or indirectly affects transcription factors such as NFkB39 [53]. Shi et al. [31] showed that Ir stimulated the proliferation of liver cancer cells under in vitro conditions by activating the PI3K/Akt pathway (Figure 2). The above study results are contrary to those obtained by Gannon et al. [30]. In turn, Moon and Mantzoros [63], in their in vitro study, showed no effect of Ir on the proliferation of endometrial, colon, thyroid, or esophageal cancer cells. Many studies support the antiproliferative effect of Ir in an in vitro model. The conflicting findings may be due to tissue and cell specificity of Ir, as reported by Shi et al. [31] (Table 1).

**Table 1:** Summary of the results of Irisin levels in in vitro model studies.

Research Team	Cancer/Cell Lines	Irisin	Results	Reference Number
Moon et al. [63]	Endometrial (KLE, RL95-2) Colon (HT29, MCA38) Thyroid (SW579, BHP7 Esophageal (OE13, OE33) KLE, RL95-2, HT29, SW579 BHP7, OE13, OE33-American Type Culture Collection (ATCC, Manassas, VA, USA) MCA38, National Cancer Institute, National Institute of Health, Dr. Nicholas Restifo	Human recombinant Ir Aviscera Bioscience (Santa Clara, CA, USA) Phoenix Pharmaceuticals (Burlingame, CA, USA) Ir levels: 5–10 nmol/L (physiological) 50–100 nmol/L (pharmacological)	No impact of Ir on tumor cell proliferation, adhesion, or number compared to controls (*p* < 0.05)	[63]
Gannon et al. [30]	Breast MCF-7 MDA-MB-231 MCF-10a- control (American Type Culture Collection; Manassas, VA, USA)	Human recombinant nonmodified Ir-INM Cayman Chemical (Ann Arbor, MI, USA) Human recombinant modified and active (glycosylated) Ir-IM PlexBio (San Francisco, CA, USA) Ir levels: 0.625–20 nM	Reduced number of cancer cells (INM), and migration (INM, IM) Induction of tumor cell apoptosis (INM) Inhibition of NF-κB activity (INM) Enhancement of the effect of Dox on cancer cells by Ir (INM at all tested concentrations; IM at 1.0 μgM)	[30]
Tekin et al. [58]	Prostate cancer LNCaP DU-145 PC3	Ir (Phoenix peptide, Burlingame, CA, USA) Ir levels: 0.1–100 nM	Antiproliferative effect Decreased survival time of LNCaP cells at higher Ir levels (10–100 nM; *p* < 0.05; *p* < 0.01)	[58]
Shi et al. [31]	Hepatocellular carcinoma HepG2 SMMC7721	Human recombinant modified and active (glycosylated) Ir-IM PlexBio (San Francisco, CA, USA) Human recombinant non-modified Ir-INM CaymanChemical (Ann Arbor, MI, USA) Ir levels: 0.625–20 nM	Ir increased liver cancer cell viability in all cell lines (IM, INM) The Ir-IM-level of 2.5 nM stimulated an increase in migration and invasiveness of HepG2 cells compared to controls. This increase was statistically significant The level of modified Ir-IM of 2.5 nM significantly inhibited the cytotoxicity of Dox	[31]
Shao et al. [59]	Lung cancer A549 (NSCLC) NCl-H446 (SCLC) Institute of Biochemistry and Cell Biology, Chinese Academy of Science, China	Ir levels: 0–50 nM	Ir at levels of 20–50 nM significantly inhibited A549 cell proliferation Ir at levels >20 nM inhibited migration and invasiveness of A549 cells	[59]
Kong et al. [61]	Osteosarcoma U2OS MG-63 American Type Culture Collection (ATCC, Manassas, VA, USA)	Ir levels: 0–200 ng/mL	Ir inhibited proliferation, migration, and invasiveness of U2OS and MG-63 cells in a dose- and time-dependent manner	[61]
Liu et al. [62]	Pancreatic cancer MIA PaCa-2 Panc03.27 ATCC (Manassas, VA, USA)	Human recombinant glycosylated E-Ir Human nonrecombinant P-Ir Sangon Biotech, Shanghai, China Ir levels: 0–100 nM	Both Ir forms inhibited the growth, migration, and invasiveness of pancreatic cancer cells	[62]

## 5. Impact of Irisin on Epithelial–Mesenchymal Transition

### 5.1. Epithelial–Mesenchymal Transition as the Background of Metastatic Processes

Malignant tumors mainly originate from the epithelial tissue, which is well structured. The cells are located on the basement membrane, form cell–cell junctions (known as desmosomes or nexus), show the expression of specific markers (i.e., E-cadherin), and are polarized [64]. Tumor cells can spread either locally through a dynamic growth or distantly. Metastasis is a multistep process that requires cancer cells to undergo changes related to motility and the ability to migrate. This process describes the phenomenon of epithelial–mesenchymal transition (EMT). Cell migration is possible due to its polarity and signaling proteins (Rho family GTPases) that influence the remodeling of the cell cytoskeleton via the formation of protrusions and the loosening of cell–cell junctions. [64] Poor oxygen conditions in the tumor increase the expression of HIF-1α, which stimulates VEGF synthesis and angiogenesis, which is possible through the activation of the PI3K/Akt pathway [65]. Cell-secreted proteases (i.e., MMP proteins) digest the extracellular matrix and allow tumor cells to penetrate the circulatory system [66]. A change in the cancer cell phenotype is the result of EMT. These cells start to resemble mesenchymal cells. The changes primarily involve the synthesis of many proteins. Surface proteins (E-cadherin) responsible for forming cell–cell junctions (integrins) are replaced with proteins responsible for migration and the loosening of these junctions. These include mesenchymal cell markers such as *N*-cadherin, vimentin, α- SMA, and the Rho proteins. Altered protein synthesis is determined by altered expression of transcription factors involved in the EMT process (Snail, SLUG, Twist) [67].

### 5.2. Results of the In Vitro Model Indicating the Impact of Irisin on Epithelial–Mesenchymal Transition

**Figure 3:**
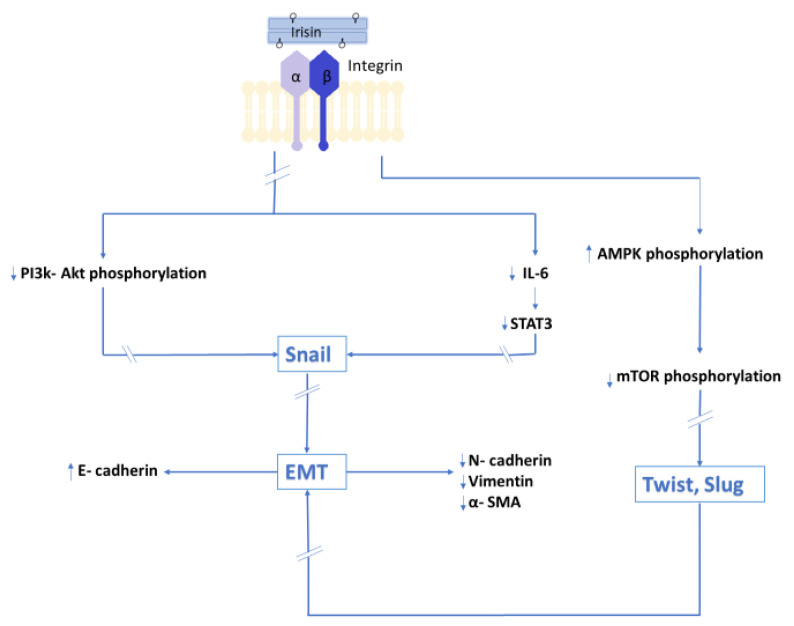
Irisin (Ir) inhibits PI3K/Akt signaling pathway and the transcription factor Snail, which is the major downregulator of E-cadherin gene expression. Ir reverses the effect of IL-6 by increasing E-cadherin expression. Ir has also an inhibitory effect on the STAT3 signaling pathway and transcription factor Snail, which are activated by IL-6 and are crucial for EMT. Ir increases the phosphorylation of AMPK and decreases the phosphorylation of the downstream molecule of AMPK signaling (mTOR pathway), which leads to the inhibition of the expression of transcription factors and the inhibition of EMT.

In an in vitro model, Shao et al. [59] showed the inhibitory effect of Ir on lung cancer cell migration. In the same study, Ir inhibited the expression of *N*-cadherin and vimentin and increased the expression of E-cadherin. In addition, the study showed the inhibitory effect of Ir on PI3K/Akt phosphorylation and the transcription factor Snail, which is the major regulator of E-cadherin that is responsible for inhibiting its gene expression. Ir alters EMT markers by inhibiting the PI3K/Akt signaling pathway in lung cancer cells, which, according to the authors, indicates its involvement in the inhibition of migration and metastasis. The inhibitory effect of Ir on EMT markers was reported by Kong et al. [61] In vitro studies showed that Ir inhibited the migration of osteosarcoma cells, thus reducing its metastatic potential. IL-6 inhibits the expression of E-cadherin. In the above study, the authors showed that Ir reversed the effect of IL-6 by increasing E-cadherin expression. However, it has an inhibitory effect on the expressions of E-cadherin, vimentin, and MMP proteins whose expression is stimulated by IL-6. In the same study, the authors also demonstrated an inhibitory effect of Ir on the STAT3 signaling pathway and transcription factor Snail, which are activated by IL-6 and are crucial for EMT of osteosarcoma. Similar results were reported by Liu et al. [62] in an in vitro model using pancreatic cancer cells. As in previous studies, Ir inhibited the migration and metastatic ability of pancreatic cancer cells. In the same study, Ir was found to inhibit the mTOR signaling pathway by activating AMPK, which is involved in the maintenance of cell energy homeostasis and is also necessary for the initiation of EMT. Different experimental results were presented by Shi et al. [31]. Under the influence of Ir, liver cancer cells increased their ability to migrate and metastasize. Moreover, Ir activated the PI3K/Akt signaling pathway. Figure 3 presents a summary of the results of in vitro studies of the influence of Irisin on EMT.

## 6. Irisin and Its Potential Role in Cancer Therapy

The effect of Ir on doxorubicin (Dox) therapy was investigated by Gannon et al. [30]. The results of this study showed the inhibitory effects of Ir on the proliferation and migration of breast cancer cells (MDA-MB-231) in an in vitro model. Dox, which can be used in breast cancer therapy, can cause many adverse effects, including cardiotoxicity. In the above study, Ir increased the cytotoxic effect of Dox, while reducing its uptake by tumor cells. Of note, Ir enhanced the cytotoxicity of Dox only in malignant cells (MCF-7) without affecting nonmalignant cells (MCF-10a). Thus, Ir may enhance the efficacy of Dox by affecting the reduction in its toxic effect on healthy cells, thus reducing the number of complications of cancer therapy. Shi et al. [31] also analyzed the effect of Ir on Dox therapy and showed that Ir reduced the cytotoxicity of Dox in liver cancer cells (HepG2). Further studies are warranted to determine whether the inconclusive study results obtained by different research groups are only related to the tissue specificity of Ir. These studies should also determine its potential role in cancer therapy.

Fan et al. [60] investigated the potential effect of Ir on paclitaxel therapy, which is used in many NSCLC treatment regimens. Silencing of FNDC5 decreased the sensitivity of NSCLC cells to paclitaxel. However, cancer cells in patients who were given Ir before treatment showed increased sensitivity to the drug, and they were characterized by higher activity of proapoptotic cells (Bax, p53) and lower levels of antiapoptotic proteins (Bcl-2). Fan et al. [60] concluded that the combined use of Ir and paclitaxel could be beneficial in the treatment of NSCLC. Moreover, it may reduce the frequent phenomenon of increasing resistance to paclitaxel in the late stage of chemotherapy.

## 7. The Role of Irisin in Selected Cancer Diseases

### 7.1. Breast Cancer and Reproductive Tract Cancer

#### 7.1.1. Serum Irisin Level in Patients with Breast Cancer

Provatopoulou et al. [13] showed lower serum Ir levels in cancer patients compared to controls (Table 2). Furthermore, it was estimated that a one unit increase in serum Ir levels resulted in the reduction in the risk of breast cancer by almost 90%. It was also shown that Ir could be a breast cancer screening marker. At a cutoff point of 3.21 μg/mL, the sensitivity and specificity were 62.7% and 91.1%, respectively. A positive correlation was found between Ir level and the clinical stage of the tumor (S). No statistically significant correlation was found with respect to tumor size (T), lymph node metastasis (N), and histological malignancy of the tumor (G). Different results were obtained by Panagiotou et al. [68]. After analyzing the results of Provatopoulou et al., [13] Panagiotou et al. [68] paid attention to different ELISA kits used by various investigators. They performed their study using ELISA, which was previously validated using mass spectrometry by Jędrychowski et al. [12]. Panagiotou et al. [68] found elevated serum Ir levels in benign and malignant breast tumors. No differences were found in Ir levels between benign and malignant tumors. However, when Ir was used with omentin-1, which is an adipokine with the properties similar to adiponectin, elevated Ir levels indicated tumor malignancy. Those authors explained different results from Provatopoulou et al. [13] by the fact that a different ELISA kit was used. Panagiotou et al. [68] reported that patients with benign breast lesions were not enrolled in the previous study. The results were explained by unknown pathophysiological phenomena occurring in malignant tumors. In addition, the authors showed a positive correlation between Ir and Ki-67, which is a marker of cell proliferation. Ir levels also increased with the grade of malignancy as described by the Elston–Ellis score and were higher in patients with a positive estrogen receptor (ER^+^). The researchers expressed the opinion that their findings indicated the possible involvement of Ir in breast tumor formation from benign lesions to malignant progression, which makes Ir a promising diagnostic and prognostic marker. In turn, Zhang et al. [15] analyzed patients with breast cancer and spinal metastasis. Their results showed that serum Ir levels were higher in patients without metastasis (M0). Furthermore, the presence of serum Ir was shown to be associated with a protective effect against the occurrence of spinal metastasis. Additionally, a positive correlation was found between serum Ir levels and BMI of patients, which could suggest that a higher amount of body fat in female patients was associated with a higher release of Ir into the serum. This suggests that the study group should be matched in terms of BMI to exclude the influence of Ir which is expressed and released from adipocytes. It may explain the opposite results when the immunohistochemical (IHC) method was used, in which Ir expression was analyzed only in the tumor tissue.

#### 7.1.2. Irisin Tissue Expression Levels in Patients with Breast Cancer and Reproductive Tract Cancer

Kuloglu et al. [73] performed IHC reactions which showed no Ir expression in normal breast tissue. However, Ir expression levels were significantly higher in invasive lobular carcinoma, intraductal papillary carcinoma, invasive ductal carcinoma, invasive papillary carcinoma, and mucinous carcinoma compared to healthy breast tissue. As opposed to the mammary gland, normal luteal cells in the ovarian region showed Ir expression. Positive IHC was found for ovarian endometrial cancer. Low Ir expression was found in mucinous ovarian cancer tissues, as well as in atypical endometrial proliferation. High levels of Ir expression were noted in benign endometrial proliferation and in cervical squamous cell carcinoma.

Further studies are warranted to determine the relationship between serum Ir levels and its expression in the tissue, as there is no available research on their relationship. The lack of knowledge about the receptor in the mammary gland tissue does not allow understanding of Ir transport from the serum to the tissue or vice versa. Different results obtained by various research teams may also be due to the lack of validation of the ELISA assays, as well as differences related to the study design and patient selection. However, the above findings indicate that Ir may be a promising biochemical marker that is a complement to screening for breast cancer. The analysis of the above studies showed that decreased serum Ir levels in women could indicate the occurrence of breast cancer and its distant metastasis.

Moreover, determination of Ir expression by IHC in biopsy material may be helpful to determine the occurrence of not only breast cancer, but also cancers of the reproductive tract. However, it is necessary to examine the relationship between Ir expression in the cancer tissue and clinicopathological factors, as was done in the case of its serum levels. Studies also indicate potential protective effects of Ir and the possibility of its use in targeted therapy.

### 7.2. Irisin in Prostate, Kidney, and Bladder Cancer

Aslan et al. [70] compared serum Ir levels in prostate cancer patients and healthy male subjects. Prostate-specific antigen levels (PSA) were significantly higher in prostate cancer patients compared to controls. The opposite trend was found in the case of serum Ir levels in cancer patients in whom these levels were decreased compared to healthy male subjects. However, no differences were found in Ir levels in patients from different groups based on the Gleason classification. The results of the above study indicated that Ir could be a useful diagnostic biomarker, which could be used as an adjunct to diagnosis using PSA.

Esawy and Abdel-Samd [71] investigated serum Ir levels in bladder cancer (BC) patients. They showed lower serum Ir levels in patients with BC compared to the control group. At the cutoff level of Ir ≤ 1.2 μg/mL, the sensitivity and specificity were 74.7% and 99.7%, respectively. No differences were found in the concentrations of biochemical parameters, i.e., fasting glucose, triglycerides, HDL, and LDL cholesterol in BC patients except for total cholesterol, which was significantly lower in the patient group. Ir levels correlated positively with BMI of patients and negatively with cholesterol levels. The association between Ir levels and grades of histological differentiation (G) was also investigated. Ir level was significantly lower in G3 compared to G1 and it also significantly decreased in the subsequent clinical stages of BC (S). The 1 year mortality rate in patients with high Ir levels was 5% compared to 38.2% in patients with low Ir levels. Patients with high Ir levels had significantly higher overall survival (OS) rates than patients with low Ir levels. The authors of the study concluded that Ir could be both a helpful marker in the diagnosis of BC and could also act as a prognostic factor for the survival of BC patients.

Altay et al. [14] showed higher serum Ir and carcinoembryonic antigen (CEA) levels in renal cancer patients compared to controls. Ir level had higher sensitivity and specificity compared to CEA level, which is a recognized cancer marker of endodermal and ectodermal origin. The authors of the study were cautious in formulating a thesis about the usefulness of Ir in the diagnosis of renal cell carcinoma. The study was conducted on a small patient population (*n* = 23). This may be the reason why those researchers had different results from those obtained in other urinary tract cancers.

Further studies are warranted on larger patient populations to confirm the results. A study on a much larger patient population was conducted by Kuloglu et al. [74] who analyzed kidney cancer tissues using IHC. Ir was not found in clear cell or papillary renal cell carcinomas. Significantly decreased Ir levels were noted in chromophobe renal cell carcinoma samples. No differences were found in the level of Ir expression in benign oncocytoma than healthy tissues, which indicates that investigation of Ir expression levels may be a useful test for differentiating benign lesions from renal cancer.

The above studies support the thesis about the usefulness of Ir in the diagnosis of urinary tract cancers. Investigation of serum Ir levels may support the diagnosis as an adjunct to the assessment of PSA level in prostate cancer and CEA level in renal cancer. In addition, the study results indicated a protective effect of Ir and higher mortality rates in patients with decreased Ir levels. This molecule can be used as a prognostic factor in BC. Studies on tissues and serum are available only in the case of renal cancer. Studies on material obtained from the same patients are necessary due to the inverse relationship (i.e., elevated serum Ir levels and decreased Ir expression in renal cancer tissue). So far, only the assessment of Ir expression using IHC has seemingly been useful for renal cancer differentiation.

### 7.3. Irisin in Gastrointestinal Cancers

Zhu et al. [69] compared serum Ir and the activating transcription factor (ATF3) levels in patients with colorectal cancer (CRC) who were overweight, obese, or of normal weight. Patients with normal weight had lower Ir levels than controls. No differences were found in serum Ir levels or FNDC5 mRNA expression in the adipose tissue of CRC patients with normal or abnormal weight. However, higher serum ATF3 levels were found in patients with normal and abnormal body weight. Moreover, Ir levels were positively correlated with triglyceride levels in CRC patients and controls. After adjusting for age, sex, BMI, and other biochemical parameters, high Ir levels reduced the risk of developing CRC by 78%, whereas high ATF3 levels increased the risk of developing this cancer. At the cutoff value for 0.46 ng/mL for ATF3, the sensitivity and specificity for the discrimination of CRC were 74% and 65%, respectively. At the cutoff value of 0.19 μg/mL for Ir, the sensitivity and specificity for the discrimination of CRC were 63% and 65%, respectively. When ATF3 and Ir were included in the analysis, the sensitivity and specificity were 73% and 80%, respectively. Therefore, combining ATF3 and Ir in the diagnostic process may increase the accuracy of CRC diagnosis.

Pazgan-Simon et al. [72] examined serum Ir and betatrophin levels in patients with cirrhosis and HCC. Ir levels were decreased in patients with HCC, whereas no significant differences were found in patients with cirrhosis compared to controls. However, betatrophin levels were higher in HCC and cirrhosis patients compared to the control group. Moreover, Ir levels were significantly decreased in more advanced stages of HCC (A vs. C according to the Barcelona Clinic Liver Cancer (BCLC)) and more advanced stages of cirrhosis (A vs. B according to the Child–Pugh score (C–P score)). The above studies indicated that Ir could have a protective effect, and its low level promotes faster fibrosis and tumor progression.

In gastrointestinal cancers, Ir was lower in the analyzed papers except for one study. Shahidi et al. [75] reported higher serum Ir levels in gastric cancer (GC) patients compared to controls. According to these authors, Ir could be a valuable biomarker in the early detection of GC. However, the small sample size was the limitation of the study. In turn, the findings of Shahidi et al. [75] are in line with the analysis of Ir levels in tissue material conducted by Aydin et al. [76] The researchers performed Ir detection with IHC using tumor fragments from patients with gastrointestinal cancers which were compared to healthy tissues. Aydin et al. [76] also found positive IHC reactions for Ir detection in healthy gastric, esophageal, colon, hepatic, and pancreatic tissues. They observed higher Ir expression in gastric adenosquamous adenocarcinoma, gastric neuroendocrine carcinoma, and gastric signet-ring cell carcinoma. The expression of Ir in gastric signet-ring cell carcinoma was higher than that in gastric neuroendocrine carcinoma and gastric adenosquamous adenocarcinoma. This cancer is characterized by a significant presence of mucin in the cell cytoplasm [77]. However, Altay et al. [57] did not demonstrate Ir expression in healthy gastric tissues or experimentally induced GC in mice. However, their study showed higher Ir levels in WAT and BAT in GC mice compared to controls and an increase in Ir expression in both adipose tissues with cancer progression.

Furthermore, the increase in Ir levels in the adipose tissue corresponded to the increase in serum Ir levels in diseased mice. Perhaps a similar mechanism also occurs in humans, which could explain the results obtained by Shahidi et al. [75] in patients with GC. The mechanism of the relationship between cancer and the increase in FNDC5 mRNA expression in the adipose tissue and the increase in serum Ir levels is unknown. The authors of this study suggested that the autocrine effect of Ir on the adipose tissue may result in weight loss in diseased mice and underlie cancer cachexia.

Aydin et al. [76] also analyzed other types of gastrointestinal cancers. They found that Ir expression in esophageal epidermoid carcinoma, esophageal adenocarcinoma, and esophageal neuroendocrine carcinoma was higher than in the healthy tissue. The level of Ir was not significantly different in various histological types of esophageal cancer. The researchers also demonstrated increased Ir expression in colon adenocarcinoma and colon mucinous adenocarcinoma. The intensity of the IHC reaction was similar for these two cancers. In intralobular and interlobular ducts of cancerous pancreatic tissue, Ir expression was also higher than in the healthy tissue. However, the results related to HCC are inconclusive. Although the healthy liver tissue showed the presence of Ir in hepatocytes, the researchers found no difference between Ir expression levels in HCC and healthy tissue.

Different study results were obtained by Zhang et al. [78]. They demonstrated decreased FNDC5 mRNA expression in tissues obtained from HCC patients, as well as decreased serum Ir levels in patients before hepatectomy. However, Gaggini et al. [56] showed overexpression of FNDC5 mRNA in HCC patients. However, no differences were found between serum Ir levels in HCC patients and healthy controls. Similar results were obtained by Shi et al. [31], who found an increase in *FNDC5* gene expression in HCC tissues and no differences between serum Ir levels in HCC patients and healthy controls. According to Gaggini et al. [56], no relationship between *FNDC5* gene expression in the tumor tissue and the protein level is very common and could be related to post-transcriptional and post-translational events (such as protein half-life, protein damage, or degradation). Recent studies have also indicated that the liver and kidney may be involved in the clearance and metabolism of Ir.

The above studies demonstrate a potential role of Ir in the diagnosis of gastrointestinal cancers. Most studies indicate an increased lr level in tumor tissues compared to healthy tissues in patients. However, there have been no studies on its association with clinicopathological factors. Studies on a mouse model indicated no relationship between the expression of Ir protein in the tissue and its serum levels. However, there are no such studies on patient material. Contradictory results are related to hepatic cancers. They may be associated with the participation of the liver in Ir metabolism. Further studies are warranted to clarify these issues. They should consider larger patient populations and critical remarks related to methodology and patient selection for studies. Differences in circulating serum Ir levels may be related to the fact that it is released by many tissues (muscle tissue, adipose tissue), which was confirmed by a study using a mouse model. Moreover, the final Ir level may depend on systemic or local expression. Another reason which has already been indicated in this paper may be the presence of monoclonal antibodies used in ELISA kits, whose sensitivity and specificity have been questioned in many studies [7,11,56].

### 7.4. Irisin in Lung Cancer

To date, only two studies have been conducted on lung cancer, including one using an in vitro model only. However, Nowinska et al. [51] conducted a study on a significantly larger population (*n* = 729) than other studies related to different cancer types. The results of their study showed Ir expression in NSCLC cells and stromal cells. Expression of Ir in the stromal cells has not been reported in any other type of cancer. This can be characteristic only of lung cancers. Ir levels were higher in cancer cell cytoplasm and stromal cells in adenocarcinoma compared to squamous cell carcinoma. Higher expression of FNDC5 mRNA in NSCLC tissues was confirmed by molecular studies (RT-PCR). Using laser capture microdissection (LCM), cancer cells and tumor cells were very precisely collected. It was shown that the expression of FNDC5 mRNA from NSCLC tissues in stromal cells was higher than that in cancer cells. No Ir expression was found in normal lung tissue except for lung macrophages. In addition, Nowinska et al. [51] showed an association between Ir expression levels in tumor cells and clinicopathological parameters. In tumor cells, Ir expression levels were decreased with higher grades (G) of malignancy and in larger tumors (T). Changes in Ir expression were also observed in relation to lymph node metastases. The expression level of Ir in tumors with mediastinal lymph node metastasis (N2) was higher than that in the group without lymph node metastasis (N0) and in the group with hilar and mediastinal lymph node metastases (N1). No association was found between Ir expression in tumor cells and overall survival (OS). Those researchers also analyzed the relationship between Ir in tumor stromal cells and clinicopathological parameters. The level of Ir expression increased in advanced pT status. They also showed a positive correlation between Ir expression in stromal cells and the level of Ki-67 antigen in cancer cells. Those authors suggested that Ir expression in stromal fibroblasts could influence NSCLC cell proliferation. This is also supported by shorter survival of patients with higher Ir expression in NSCLC stromal cells. Higher Ir expression was demonstrated in patients with distant metastases (M1) compared to nonmetastatic patients (M0). Furthermore, the study results confirmed that Ir expression in stromal cells might be an independent prognostic factor.

The role of Ir in lung cancer has not yet been fully understood. Nowinska et al. [51] were the first to describe Ir in the context of lung tissue and lung cancer. An earlier study by Shao et al. [59], who used an in vitro model, demonstrated the inhibitory effect of Ir on lung cancer cell proliferation, migration, and invasion by inhibiting the PI3K/Akt pathway. Furthermore, Ir can reverse the EMT process by inhibiting the expression of the transcription factor Snail. Studies using tissues collected from lung cancer patients also indicated its association with cell proliferation and lymph node and distant metastases. However, the mechanism present in the tissues obtained from patients appears to be more complicated due to a significant impact of Ir expression in the tumor stroma on disease progression. The authors suggested that high Ir levels in cancer cells during the first stage of the disease could be related to changes in their metabolism and mitochondrial biogenesis. However, in later stages of the disease, Ir expression may be inhibited due to the impact of Ir on UCP1 expression and ATP synthesis reduction. A decreased ATP level is associated with activation of AMPK and inhibition of the mTOR pathway. The AMPK–mTOR pathway plays an important role in cell proliferation. However, further studies are warranted to explain the mechanism of how Ir affects cancer cells. Additionally, there have been no studies related to the assessment of serum Ir levels in patients with lung cancer.

### 7.5. Irisin in Thyroid Cancer

Ugur et al. [79] conducted a study on different histological types of thyroid carcinomas and compared them to healthy tissue. Ir expression was assessed using IHC, and Ir levels were measured using ELISA. Tissue samples were homogenized. Ir expression was slightly increased in patients with papillary thyroid carcinoma (PTC) and significantly increased in oncocytic papillary thyroid carcinoma (OPTC) and anaplastic thyroid carcinoma (ATC). However, no differences were found in follicular thyroid carcinoma (FTC). Ir expression was higher in the tissues of patients with oncocytic follicular thyroid carcinoma (OFTC) than in FTC. No Ir immunoreactivity was found in the tissues of patients with medullary thyroid carcinoma (MTC). The assay using ELISA confirmed the IHC results. Those authors indicated that most oncocytic follicular cells have a structure similar to Hürthle cells (HCs).

On the other hand, HC metaplasia is an important feature of chronic lymphocytic thyroiditis (Hashimoto’s thyroiditis; HT) [80]. HCs have many mitochondria and are associated with energy production. Ugur et al. [79] demonstrated increased Ir expression in oncocytic carcinomas and tissues obtained from HT patients. The researchers concluded that the thyroid tissue rich in HC (in other words, rich in mitochondria) produced more heat and caused the death of oncocytic cells, which occurred due to increased Ir levels in oncocytic tumors and increasing concentration of UCP1 in mitochondria. PTCs and FTCs with fewer mitochondria and lower Ir expression generated less heat and were perhaps more clinically aggressive than oncocytic variants. Additionally, Ugur et al. [79] found suppressed Ir expression in MTCs. The more aggressive course of these cancers may be due to decreased Ir synthesis, which may show a protective effect, as reported by those authors. Ir is a promising biomarker which is useful for differentiating oncocytic variants of OPTC and OFTC from non-oncocytic forms of PTC and FTC. Ir may mediate thyroid carcinogenesis and participate in oncocytic cell apoptosis through increased heat production.

### 7.6. Osteosarcoma

**Figure 4:**
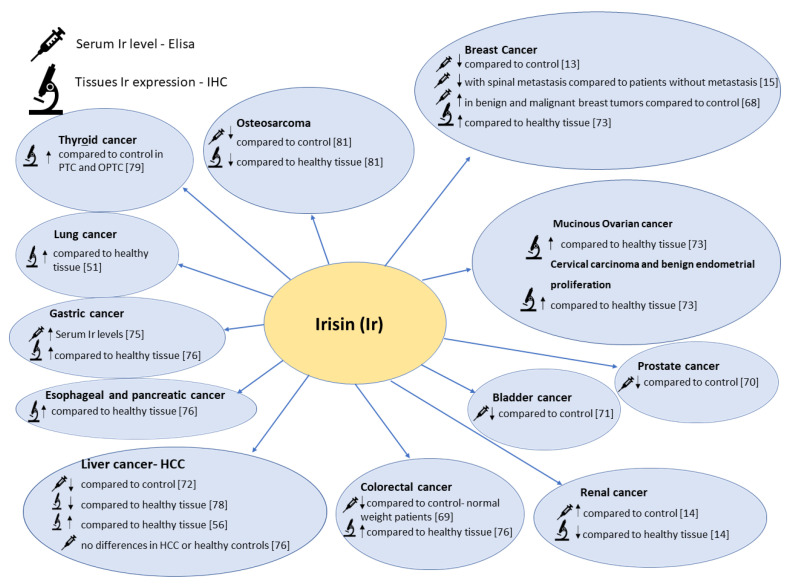
The figure shows the comparison between serum Irisin levels and IHC Irisin expression levels in different types of cancers.

Cheng et al. [81] showed a decreased level of FNDC5/Ir in serum and tissues of osteosarcoma patients. The researchers also performed in vitro studies using osteosarcoma U2OS cell lines and found that Ir inhibited U2OS cell viability in a concentration- and time-dependent manner and could inhibit tumor cell migration and invasion. The microRNA (miR) 214-3p was also used in this study. MicroRNAs (miRNA/miR) are small noncoding RNAs involved in tumor initiation, growth, and progression. In previous studies, miR-214 was upregulated in osteosarcoma cells and was associated with tumor progression and poor prognosis [82]. Cheng et al. [81] found that miR-214-3p inhibited FNDC5/Ir expression, thus contributing to the activation of migration, invasion, and EMT of osteosarcoma cells (Figure 4). Previous studies showed that Ir activated osteocytes through the αV/β5 integrin receptor [36].

Osteocytes control skeletal remodeling by inducing osteoclastogenesis and inhibiting osteogenesis. Kim et al. [36] showed that deletion of *FNDC5* inhibited bone resorption by blocking the increase in the number of osteoclasts, thus preventing the loss of bone mass. Other findings were obtained by Colaianni et al. [33] who reported that Ir which was given to mice with osteoporosis prevented bone mass loss and induced bone regeneration. Moreover, it was found that Ir protected against the loss of muscle mass in immobilized mice. Kim et al. [36] suggested that Ir could affect bone resorption and remodeling when dosed appropriately. Parathormone (PTH) shows a similar effect. Peritumoral osteolysis is essential in the development of osteosarcoma. Osteoclast activity leads to bone degradation and the release of protumor factors, such as insulin-like growth factor 1 (IGF1) or transforming growth factor-β (TGF-β), which enhance osteosarcoma cell proliferation [83]. Cheng et al. [81] indicated a protective role of Ir in the proliferation and metastasis of osteosarcoma. These authors indicated that Ir could be used for osteosarcoma therapy in the future.

## 8. Conclusions

Irisin has been described relatively recently in the literature as a myokine released by skeletal muscles under the influence of physical exercise. It soon became evident that Ir expression is also present in other tissues, including normal and abnormal tissues such as cancer tissues. Many studies have been conducted to determine the role of Ir in both physiological and pathological processes.

Studies on animal models and cell lines are underway. They are related to the role of Ir in cancer disease, including many types of malignancies, i.e., breast, lung, gastrointestinal, reproductive tract, and bone cancers. The recent studies mostly showed an inhibitory effect of Ir on the proliferation, migration, and invasiveness of cancer cells. They also indicated the inhibitory effect of Ir on the processes related to EMT, which is crucial for cancer cell metastasis. The conflicting results found in gastrointestinal cancers are probably due to the tissue specificity of Ir, and further studies are warranted in this respect. Additionally, molecular studies which use both tumor tissues and serum of patients are also being conducted. Serum Ir levels are different in cancer patients. They decrease or increase in patients with breast cancer, increase in patients with renal cancer, and remain stable in patients with liver cancer. The observed differences may be due to the release of Ir by different tissues, and its level is the result of local and systemic production. It may also result from the circulation of Ir isoforms and the type of assays used to detect it. Lastly, the most important issue is related to the structure and occurrence of the receptor for Ir. Additionally, cancer stromal cells show the expression of Ir. Their role in promoting tumor proliferation seems particularly interesting. Ir also shows therapeutic potential. However, further studies are warranted to determine its effect on Dox therapy.

To conclude, many studies have been conducted to determine the role Ir plays in the body in health and disease. Small sample sizes, the lack of correlations between the study results and clinicopathological factors, and studies using only animal models or in vitro experiments were some of the identified limitations, as admitted by the researchers themselves. Comprehensive studies defining the role of Ir in pathological processes and in the development of cancer could show its real usefulness in disease prevention, diagnosis, and treatment.

## Figures and Tables

**Table 2:** Summary of the study results of Irisin levels in human plasma detected by ELISA.

Research Team	Study Method	Results	Study Group	Reference Number
Provatopoulou et al. [13]	ELISA (AdipoGen International, Liestal, SW); results expressed as μg/mL	Lower serum levels of Ir in patients compared to the control group (2.47 ± 0.57 (mean ± SD) vs. 3.24 ± 0.66 (mean ± SD)) *p* < 0.001	101 female patients with invasive ductal breast cancer 51 healthy women (the control group)	[13]
Gaggini et al. [56]	ELISA (Adipogen AG, Liestal, Switzerland); results expressed as μg/mL	Plasma Ir levels did not differ between HCC patients and controls (3.56 ± 0.2 (mean ± SEM) vs. 4.4 ± 0.15 (mean ± SEM)) *p* = 0.749	18 patients with HCC 18 deceased donors	[56]
Shi et al. [31]	ELISA (USCN life Science, Wuhan, China); results expressed as μg/mL	Plasma Ir levels were not different between HCC patients and controls	20 patients with HCC	[31]
Altay et al. [14]	ELISA (USCN, Life Science Inc., Catalog No. USCN-E82576Hu, P.R. China); results expressed as pg/mL	Higher FNDC5/Ir levels in renal tumor patients compared to the control group (208 ± 97 (mean ± SD) vs. 110 ± 79 [mean ± SD) *p* = 0.0001	23 patients with renal tumor 25 healthy individuals	[14]
Zhang et al. [15]	ELISA (Aviscera Biosciences, Santa Clara, CA, USA); results expressed as ng/mL	Higher Ir levels in patients without spinal metastases (7.60 ± 3.80 (mean ± SD) vs. 6.10 ± 2.62 (mean ± SD)) *p* = 0.012	148 patients with breast cancer, including 53 subjects with spinal metastasis	[15]
Zhu et al. [69]	ELISA (USCN Life Science Inc., Wuhan, China); results expressed as μg/mL	Lower Ir levels in patients with colorectal cancer and normal weight compared to controls (0.17 ± 0.01 (mean ± SD) vs. 0.22 ± 0.01 (mean ± SD)) *p* < 0.05)	76 patients—38 patients with colon cancer and 38 subjects with rectal cancer 40 healthy controls	[69]
Aslan et al. [70]	ELISA (Yl Biont Biotech Co. Shanghai, China); results expressed as pg/mL	Mean Ir level was lower in prostate cancer patients compared to controls (6.92 ± 2.44 (mean ± SD) and 13.5 ± 6.21 (mean ± SD)) *p* < 0.05	50 patients with primary prostate cancer 30 healthy male subjects	[70]
Esawy and Abel [71]	ELISA (Bio Vendor Laboratory Medicine, Brno, Czech Republic) [Catalog No. RAG018R]; results expressed as μg/mL	Lower Ir levels in patients with bladder cancer compared to controls (1.07 (0.51–1.96) (mean ± SD) vs. 1.8 (0.5–2.44) (mean ± SD)) *p* < 0.001	75 patients with bladder cancer 75 healthy subjects	[71]
Pazgan-Simon et al. [72]	ELISA (Bio Vendor- Laboratorini Medicina a.s. catalog No. RAG018R); results expressed as μg/mL	Lower Ir levels in HCC patients compared to controls (2.52 ± 1.14 (median ± SD) vs. 4.46 ± 1.34 (median ± SD)) *p* = 0.02	69 patients with cirrhosis and hepatocellular carcinoma 24 patients with non-viral cirrhosis 20 healthy volunteers	[72]

SD—standard deviation, SEM—standard error of mean.

## Abbreviations

Ir	Irisin
FNDC5	fibronectin type III domain-containing protein 5
WAT	white adipose tissue
BAT	brown adipose tissue
UCP	uncoupling protein
UCP1	uncoupling protein 1
PPAR	peroxisome proliferator-activated receptor
PGC1α	peroxisome proliferator-activated receptor gamma coactivator 1 alpha
FAK	focal adhesion kinase;
BMP-7	bone morphogenetic protein 7
CAFs	cancer-associated fibroblasts
GLUT	glucose transporters;
HIFs	hypoxia-inducible factors
VEGF	vascular endothelial growth factor
NF-κB	nuclear factor kappa-light-chain-enhancer of activated B cells
Akt	protein kinase B (PKB)
PI3K-3	phosphatidylinositol 3-kinase;
mTOR	mammalian target of rapamycin kinase
AMPK	5’AMP-activated protein kinase
MMP	matrix metalloproteinase
EMT	epithelial–mesenchymal transition
α-SMA	alpha-smooth muscle actin
NSCLC	non-small-cell lung carcinoma
SCLC	small-cell lung carcinoma
DOX	doxorubicin
INM	human recombinant nonmodified irisin
IM	human recombinant modified and active (glycosylated irisin)
HCC	human hepatocellular carcinoma;
DCIS	ductal carcinoma in situ
CEA	carcinoembryonic antigen
RCC	renal cell carcinoma
ccRCC	clear cell renal cell carcinoma
CRC	colorectal cancer
OS	overall survival
PTC	papillary thyroid carcinoma
FTC	follicular thyroid carcinoma
OPTC	oncocytic variant of papillary carcinoma of the thyroid
OFTC	oncocytic variant of follicular carcinoma of the thyroid;
BC	bladder cancer
GC	gastric cancer
